# GRK2 and Mitochondrial Dynamics in Cardiovascular Health and Disease

**DOI:** 10.3390/ijms26052299

**Published:** 2025-03-05

**Authors:** Cristina Gatto, Maria Rosaria Rusciano, Valeria Visco, Michele Ciccarelli

**Affiliations:** 1 Department of Medicine, Surgery and Dentistry, University of Salerno “Scuola Medica Salernitana”, 84081 Baronissi, Italy; crgatto@unisa.it (C.G.); mrusciano@unisa.it (M.R.R.); vvisco@unisa.it (V.V.); 2 Scuola di Specializzazione in Patologia Clinica e Biochimica Clinica, University of Salerno “Scuola Medica Salernitana”, 84081 Baronissi, Italy

**Keywords:** GRK2, cardiovascular disease, mitochondria, cardiovascular health

## Abstract

G protein-coupled receptors (GPCRs) represent a family of membrane proteins that regulate several cellular processes. Among the GPCRs, G protein-coupled receptor kinases (GRKs) regulate downstream signaling pathways and receptor desensitization. GRK2 has gained significant interest due to its cardiovascular physiology and pathological involvement. GRK2’s presence in cardiac tissue and its influence on cardiac function, β-adrenergic signaling, and myocardial remodeling underlies its involvement in cardiovascular diseases such as heart failure and ischemia. GRK2’s canonical role is receptor desensitization, but emerging evidence suggests its involvement in mitochondrial dynamics and bioenergetics, influencing processes such as oxidative phosphorylation, reactive oxygen species production, and apoptosis. Moreover, GRK2’s localization within mitochondria suggests a direct role in regulating mitochondrial health and function. Notably, while GRK2 inhibition seems to be a therapeutic approach to heart failure, its precise role in mitochondrial dynamics and pathology needs further investigation. This review explores the complex relationship between mitochondrial function and GRK2 and clarifies the implications for cardiovascular health. Cardiovascular medicine might greatly benefit from future studies that focus on understanding the processes behind GRK2–mitochondrial crosstalk to develop personalized therapies

## 1. Introduction

Humans can respond to external stimuli thanks to the presence of receptors. Many cellular responses to hormones and neurotransmitters are mediated by the GPCR family of membrane proteins, which is also in charge of taste, smell, and vision [1]. Since they regulate a large part of the downstream cellular response, they have aroused great interest in the field of pharmacology and could be a valid target for pharmacological compounds [2].

GPCRs have seven membrane-spanning helical segments, which are divided into parts by alternating intracellular and extracellular loop sections. G protein-coupled receptor kinases (GRKs), a family of seven serine/threonine protein kinases, identify and phosphorylate agonist-activated GPCRs [3]. GRK-mediated receptor phosphorylation is a well-known process for desensitizing GPCRs. The receptor becomes phosphorylated, allowing arrestins to bind and block the activity of G proteins, leading to rapid homologous desensitization [4]. Based on their structural and sequence similarity, the GRK family members can be divided into three major groups: the visual GRK subfamily (GRK1 and GRK7), the b-adrenergic receptor kinase subfamily (GRK2/GRK3), and the GRK4 subfamily (GRK4, GRK5, and GRK6) [5,6]. GRK 2, 3, 5, and 6 are ubiquitously expressed in mammalian tissues, while GRK1 and 7 are expressed in different species in both rods and cones and promote rhodopsin desensitization in the rod cell [7].

Among them, the one-of-a-kind Ser/Thr protein kinase G protein-coupled receptor kinase 2 (GRK2) is most known for its role in the rapid desensitization of GPCRs [8]. GRK2 was first discovered to phosphorylate the β2-adrenergic receptor. Since then, it has been thoroughly investigated as a regulator of adrenergic receptors in the heart [9]. Lately, GRK2 has emerged as a key player in the intricate web of signaling pathways within cardiac tissue.

GRK2 is the main isoform expressed highly in the myocardium and endothelium, and it is involved in heart development [10,11] and β-adrenergic signaling. The structural architecture of GRK2 is composed of a central catalytic domain, an N-terminal region including an RH domain (regulator of G protein signaling (RGS) homology domain), and a C-terminal domain, which have been demonstrated to regulate membrane location and signal transduction [12]. Numerous biological and physiological processes, including metabolic homeostasis, angiogenesis, vasodilatation, cellular migration, cardiac contractility, cellular proliferation, and cell cycle regulation, are due to GRK2 activity, so it was proposed that the changes in GRK2 levels and functions in a variety of clinical situations play a crucial role in the onset of diseases [6,13]. Particularly, it has been observed that increased protein expression is a marker of cardiac damage and heart failure (HF) [14].

Ramos Ruiz et al. discovered that the circumstances that lead to physiological vasoconstriction and hypertrophy significantly increase GRK2 activity in aortic smooth muscle cells [15]. In contrast, pro-inflammatory cytokines induce the reverse effect, suggesting that the interaction of different signal transduction pathways highly modulates the transcriptional level of GRK2 [3].

The review aims to explore the specific role of GRK2 in cardiovascular health and disease, with a primary focus on its impact on mitochondrial function. Understanding the interplay between GRK2 and mitochondria holds significant implications for exploring the complexities of cardiovascular physiology and pathology. Targeting GRK2–mitochondrial interactions may offer new therapeutic targets for pharmacological strategies.

## 2. GRK2 Structure and Function

GRK2 is a unique member of the GRK family [16]. Its structural architecture is similar to that of the other GRKs; briefly, it was demonstrated that the N-terminal domain is essential for the identification of the receptor and anchoring of the intracellular membrane. Moreover, the N-terminus includes the RH domain, which regulates GPCR signaling independently of phosphorylation and a Gβγ-binding site, which contributes to the binding of GRK2 to the membrane [17]. The C-terminal domain provides cellular localization and agonist-dependent translocation. It also includes the pleckstrin homology domain PH, which interacts with the membrane phospholipid PIP2 and free Gβγ subunits [18]. The central core constitutes the catalytic domain where the ATP binding site is located [18,19].

GRK2 is involved in several cellular and molecular processes, interacting with a variety of cellular partners [3]. Firstly, it was discovered for its role in inducing the phosphorylation of activated GPCRs, allowing arrestin binding and, thus, receptor desensitization [20]. This mechanism is part of GRK2’s canonical role, which is to phosphorylate the beta-adrenergic receptor by desensitizing it and preventing the stimulation of cells by catecholamines [21]. However, GRK2’s role was extended since it was demonstrated to be involved in other signaling mechanisms that include receptors, like IGF-1, insulin, PDGF, or EGF [22], or non-GPCR, non-plasma membrane receptor substrates like IKappaBalpha [23] or the p38 MAPK [24]. GRK2 also interacts with various intracellular non-receptor proteins, including PI3K, Akt, and ERK, thereby influencing cellular processes such as metabolism, inflammation, and cardiovascular function [25].

Dysregulation of GRK2 expression or activity has been found to be involved in several pathological conditions, including cardiovascular disease, obesity, insulin resistance, and cancer where, GRK2 upregulation can affect β-adrenergic receptor function and exacerbate disease progression [17].

## 3. GRK2 Implications in Cardiovascular Disease

Cardiovascular (CV) disease is a general term that indicates conditions affecting the heart and blood vessels, including coronary artery diseases, HF, cardiomyopathy, arrhythmia, aortic aneurysms, peripheral artery disease, thromboembolic disease, and venous thrombosis. According to the World Health Organization (WHO), CVs are still the main cause of death in Europe and worldwide, so finding new management strategies, therapies, or pharmacological targets is one of the primary goals of medical research [26].

Angiotensin-converting enzyme inhibitors (ACEIs), angiotensin II receptor blockers (ARBs), angiotensin receptor–neprilysin inhibitor (ARNI) association, SGLT2 inhibitors, and βAR antagonists (β-blockers) are commonly used to treat CV diseases, either separately or in combination [27,28,29]. Indeed, when administrated, they reduce the incidence of death and the hospitalization rate, enhance hemodynamics and symptoms, and improve left ventricular function [30,31]. However, HF still represents one of the leading causes of disability and mortality in the world [32]; consequently, the discovery and implementation of specific novel targets may improve the tailoring of treatment for CV diseases. The GRK family could be an attractive target in this scenario due to its role in cardiac function and remodeling.

The primary subfamilies of GPCRs that mediate normal function in the heart are adrenergic (α and β) receptors, endothelin-1 receptors, and angiotensin II receptors (AT1R and AT2R). However, dysregulation of these receptors is associated with the main known cardiac diseases. Chronic cardiac β-adrenoreceptor activation (β-AR) affects downstream G protein signaling and myocardial cellular responses such as hypertension, typically accompanied by elevated levels of catecholamines in the bloodstream [33].

GRK2, traditionally known for its role in desensitizing GPCRs, has extended beyond receptor regulation. GRK2 is a multifaceted regulator in cardiac tissue, participating in various signaling cascades that modulate cardiac contractility, hypertrophy, and function. Its expression and activity levels are dynamically regulated under physiological and pathological conditions, emphasizing its importance in cardiac homeostasis [3].

A critical role for GRK2 in heart disorders is supported by multiple lines of evidence; cardiac GRK2 levels increase in human and experimental models of HF, ischemia, hypertension, and the early phases of maladaptive myocardial remodeling and lymphocytes of patients with cardiac failure [34]. Furthermore, GRK2 overexpression exacerbates the development of HF in response to ischemic stress [35]. Reduced β-adrenoreceptor responsiveness in cardiac tissues is a hallmark of HF, and chronic HF causes overexpression of GRK2, which causes β-AR phosphorylation and desensitization, suggesting the idea that GRK2 inhibition can reverse or prevent HF [13].

Hence, the inhibition of GRK2 is emerging as a treatment for HF [36], although the exact nature of the cardioprotective effects of GRK2 inhibition is still unknown.

In the last few years, research has focused on the effect of GRK2 inhibition. Alternative strategies to inhibit GRK2 activity have been attempted in different ways, such as inhibiting GRK2 activity or through GRK2 genetic ablation [37].

βARKct, a short peptide extracted from GRK2’s Gβγ binding region, can suppress GRK2 signaling, promoting its displacement from the plasma membrane necessary for GRK2 function on active receptors [38]. In a study, human cardiac progenitor cells (hCPCs), isolated from patients undergoing left ventricular assist device implantation and engineered with βARKct, showed that GRK2 inhibition enhanced β2-AR/AKT/eNOS-mediated signaling after catecholamine stimulation and increased viability and reduced cell death under oxidative stress compared to control cells [39]. In a mouse model of post-myocardial infarction HF, it was found that treatment with the selective serotonin reuptake inhibitor paroxetine may inhibit GRK2 and promote βAR-mediated cardiomyocyte contractility [40]. A GRK2 increase can also impact mitochondrial function [41], glucose metabolism in the myocardium [42,43], and oxide nitric signaling [35,44] negatively, so its inhibition may enhance cardiac function and remodeling through a variety of pathways. Research has demonstrated that GRK2 deletion enhances mitochondrial biogenesis and protects against mitochondrial fragmentation, leading to better energy metabolism and cell survival [45].

However, even though an increase in GRK2 is linked to cardiac pathologies, it is essential for embryonic development. Its global knockout in mice is associated with embryonic lethality, and GRK2-KO embryos show severe abnormalities [10,46].

**Figure 1:**
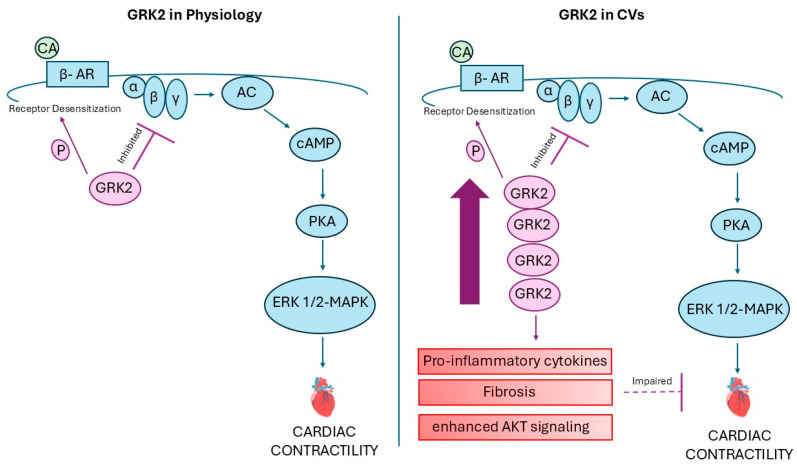
The role of GRK2 in cardiovascular disease. βAR—β-adrenergic receptor; CA—catecholamines; AC—adenylate cyclase; PKA—protein kinase A.

Research in vivo on mice with selective deletion of GRK2 shows protection against adverse remodeling [11]. A study on the myocardial-specific effects of GRK2 ablation in cardiac fibroblasts following ischemic injury details its long-term advantages for heart function as it prevents myocyte death and excessive inflammation [47]. Additionally, cardiac fibrosis and inflammation significantly affect heart failure progression. GRK2 inhibition decreases pro-inflammatory cytokines and fibrosis, leading to improved cardiac function. This is partly due to the modulation of pathways such as PI3K/Akt and ERK signaling, which are influenced by GRK2 activity [48]. The study conducted by Schlegel et al. found increased GRK2 mRNA and protein levels following transverse aortic constriction in mice. In isolated neonatal rat cardiac myocytes, stimulation with angiotensin II and phenylephrine enhanced GRK2 expression, leading to enhanced signaling via protein kinase B (PKB) or Akt, inhibiting glycogen synthase kinase 3 beta (GSK3β), promoting nuclear accumulation and activation of the nuclear factor of activated T-cells (NFAT). Cardiac myocyte hypertrophy was induced by GRK2 overexpression and the inhibition of PI3Kγ. SiRNA-mediated GRK2 knockdown prevented Akt activation, arresting NFAT activity and thus reducing cardiac myocyte hypertrophy [14] (Figure 1).

GRK2 was initially found to be a cytosolic protein that can move to the plasma membrane in response to activation from GPCRs [16,49]. Despite its cytosolic localization, GRK2 can localize into mitochondria under different stimuli, which can be considered a pro-death kinase [41]. Increased GRK2 levels induce an impairment of the cardioprotective eNOS pathway and reduced oxide nitric availability; thus, it can be considered a hallmark of cardiac damage [44].

## 4. Mitochondrial Dynamics and Function

Mitochondria are known as the powerhouses of cells and play a pivotal role in energy production, calcium homeostasis, and apoptosis, as well as functioning as a hub of several metabolic pathways, including the tricarboxylic acid cycle and fatty acid oxidation [50]. Their primary role is the production of ATP through oxidative phosphorylation, where electron flux, from complexes I to IV, is essential for membrane potential and ATP synthesis, but also contributes to the production of reactive oxygen species (ROS) [51]. An increase in ROS production can be due to external sources, such as ultraviolet rays, or internal sources as an alteration in the electron transport chain or an alteration in the antioxidant system (superoxide dismutase, catalase, glutathione peroxidase), leading to a condition known as oxidative stress that can damage mitochondria themselves [52].

Mitochondria are dynamic organelles, and their functional states are regulated by several mechanisms such as biogenesis, fusion, and fission, and a huge number of proteins activated in different cell states [53]. Cellular health is directly impacted by the complex balance between the fusion and fission dynamics of the mitochondria. Mitochondria are generated through biogenesis and fusion processes.

Biogenesis requires the synthesis of mtDNA, proteins, and membranes, and peroxisome proliferator-activated receptor gamma coactivator 1 (PGC-1α) is the master regulator of mitochondrial biogenesis. Indeed, under different stimuli, PGC-1α translocates from the cytosol to the nucleus, where it coactivates various transcriptional factors (PPARα, NRF, TFAM) [54].

Fission is a process whereby mitochondria are divided in two, generating new mitochondria from existing ones. The fission process is mediated by dynamin-related protein 1 (Drp1), which is cytosolic but recruited to the mitochondrial surface to catalyze mitochondrial fission [55].

The quantity and functional status of mitochondria in the cells are mediated by fusion and mitophagy. The fusion process requires the fusion of the outer membrane, mediated by Mitofusin 1 and 2 (MFN-1, MFN-2), and the inner membrane, mediated by optic atrophy 1 (OPA1), to allow the transfer of gene products between mitochondria [56]. Mitophagy, a term introduced by Lemasters to describe a process where mitochondria are destroyed within lysosomes through the PINK/Parkin system during mitochondria turnover or pathological situations [57], is the mechanism used to remove damaged or depolarized mitochondria. An alteration in the electron transport chain and ATP synthesis, a lack of appropriate substrates for mitochondria, or an insufficient number of mitochondria can cause mitochondrial dysfunction [58].

Mitochondrial dysfunction is one of the main pathological mechanisms in metabolic, age-related diseases, neurodegenerative disease, and ischemic injury in the heart and brain, making them a focal point of research in cardiovascular biology. Dysregulated mitochondrial dynamics exacerbate cardiac dysfunction in heart disease by impairing energy generation, oxidative stress, and apoptosis [59].

One of the main features of HF is inadequate mitochondrial activity, which decreases ATP synthesis and increases ROS production. This results in a lower energy supply to the heart muscle cells, which affects contractile efficiency and accelerates the progression of HF [60]. Research has shown that a disturbance in mitochondrial fusion in mature cardiac tissue can lead to cardiomyopathy and disrupt cardiac homeostasis in mice, suggesting a direct influence of mitochondria on cardiovascular health [61]. While Drp1 mediates excessive mitochondrial fission, which is associated with cardiomyocyte death and heart failure, mitochondrial fusion proteins, including MFN-1 and MFN-2, and OPA1, control mitochondrial connectivity and ATP synthesis [62]. Furthermore, impaired mitophagy inhibits the removal of damaged mitochondria, resulting in inflammation and mitochondrial dysfunction—two characteristics of diabetic cardiomyopathy and ischemic heart disease [63].

On the other hand, improving mitochondrial efficiency and regulating mitochondrial dynamics reduce the risk of diseases and are beneficial for health [60]. In the adult heart, the mitochondrial dynamics process is slow compared to that in the young in the absence of stress. This disparity is due to different mitochondrial distribution within cardiomyocytes [64].

A growing body of evidence suggests a direct connection between GRK2 and mitochondrial function. GRK2 has been identified within the mitochondrial compartment, affects ATP synthesis, and promotes mitochondrial biogenesis, influencing processes such as oxidative phosphorylation, ROS production, and apoptosis [65].

## 5. GRK2 and Mitochondrial Crosstalk

Recent studies have highlighted the crosstalk between GRK2 and mitochondria. A wide range of factors may modulate the level of mitochondrial GRK2, and certain stressor events (inflammation or LPS stimulation) may enhance this process [66].

Obrenovich et al. demonstrated that GRK2 accumulates in damaged mitochondria in models of ischemia–reperfusion brain injury and is an early marker of Alzheimer’s Disease [67]. In tissue with high energy demand like the heart, the presence of GRK2 in mitochondria suggests an implication in energy balance [41,68]. The mechanisms underlying this interaction involve regulating key mitochondrial proteins and processes, including oxidative phosphorylation, ROS production, and apoptosis [65].

Mitochondrial GRK2 plays a role in bioenergetic remodeling, altering the flow and use of cell substrates. Research on isolated primary adult cardiomyocytes from transgenic GRK2-overexpressing mice demonstrated that GRK2 overexpression in myocytes stimulates palmitate-induced cell death. Furthermore, the upregulation of GRK2 impaired isoproterenol, a non-selectiveβAR-agonist, and mediated mitochondrial function [69]. Furthermore, research on GRK2 inhibition has established its role in mitochondrial bioenergetics. The pharmacological inhibition of GRK2 through a chronic infusion of a cyclic peptide, C7, in post-myocardial heart-failed (post-MI HF) mice showed an improvement in cardiac parameters and, at the molecular level, improved mitochondrial organization and function. GRK2 inhibition elicits considerable recovery of mitochondrial shape and attachment to cardiac fibers, normalization of the metabolic gene profile, and a reduction in lipid accumulation within the cardiac fiber. Additionally, in post-MI HF mice, it restores the expression of genes linked to the electron transport chain, mitochondrial biogenesis, and ATP production [70]. It has previously been demonstrated that the phosphorylation of GRK2 at Ser670 is necessary for GRK2 translocation into mitochondria following ischemia/reperfusion (IR) damage in vitro, and this localization induces apoptosis. After IR damage, mice with an endogenous GRK2 knock-in mutant displaying S670A displayed decreased cardiomyocyte death and improved cardiac function, improved glucose oxidation, and the preservation of pyruvate dehydrogenase activity, suggesting the idea that GRK2 negatively impacts heart glucose oxidation following injury [71].

However, GRK2’s function at the mitochondrial level has been noted beyond the heart as well, showing a possible different role. Stable myoblast lines that overexpress GRK2 showed an increased mitochondrial mass and respiration. Interestingly, the overexpression of GRK2 was unable to prevent the myostatin-mediated impairment of mitochondrial respiratory function, but elevated levels of GRK2 blocked the increased autophagic flux observed following treatment with myostatin. These results demonstrate that GRK2 has a positive function in skeletal muscle cells by promoting mitochondrial respiration, inhibiting autophagy, and preserving mitochondrial structure [72].

Oxidative stress can induce GRK2 accumulation within the mitochondrial compartment, directly influencing mitochondrial function. In ischemic conditions, GRK2 is phosphorylated on Ser670 within the carboxyl terminus by an extracellular-signal-regulated kinase (ERK), allowing GRK2 to bind heat shock protein 90, which drives the kinase to mitochondria. It appears that calcium-induced opening of the mitochondrial permeability transition pore and mitochondrial-dependent death pathway signaling depend on upregulated mitochondrial GRK2 [41].

Moreover, a study on cardiomyocytes of transgenic mice overexpressing GRK2 shows that independently of cardiac injury, so in basal conditions, GRK2 is localized in the mitochondria, and its kinase activity negatively impacts mitochondrial function, increasing superoxide levels and altering substrate utilization for energy with decreased utilization of exogenous fatty acids [68]. The study seems to be promising, paving the way to a new aspect of GRK2 function, although its findings contradict those of another study, where the knockdown of GRK2 in macrophages increases ROS levels [73]. These differences in results could be due to GRK2 overexpression in the immune system already being present in the basal condition. The ablation induces inflammation linked to mitochondrial damage and ROS production [74]. However, GRK2’s precise role in mitochondria is not entirely clear, especially if GRK2 inhibition always has a protective effect.

GRK2 localization into mitochondria can act as a protector for organelles. It is worth mentioning that a previous study using a cellular human model (HEK293) shows that exposure to acute damage, such as ionizing radiation exposure, induces mitochondrial damage in terms of mass, shape, and respiration. According to Franco et al., GRK2-silencing cells cannot fix mitochondrial damage even after several hours. At the same time, GRK2 interacts with HSP90 to play a protective role against mitochondrial damage. This interaction phosphorylates MFN-1 and MFN-2, which are involved in mitochondrial fusion and recovery, a crucial process for mitochondrial health [75].

Moreover, the endothelium seems to be negatively impacted by GRK2 genetic ablation that compromises vascular integrity and function. The degeneration of collagen and elastic fibers, abnormal vasoconstriction, and increased lipid accumulation in the aorta wall of mice with a deletion of GRK2 in the endothelium through the CRE-plox system (Tie2CRE-GRK2fl/fl mice) indicate that the absence of GRK2 from the endothelium is associated with an inflammatory condition. Since GRK2 can localize into mitochondria and regulate ATP production and biogenesis, it was demonstrated that the removal of GRK2 increases ROS production, which can be restored by chronic treatment with a reactive oxygen species scavenger [76].

GRK2 is a serine/threonine kinase known for its involvement in cell regulation. Its translocation into the mitochondria introduces a new aspect of its activity and indicates its involvement within these organelles as well. The nature of its activity remains unclear, and the disparity of data obtained in various studies could be due to the different study models used, the different cell types, and the different kinds of stimuli (Table 1). Further studies standardizing the conditions used appear to be necessary.

The main findings of these studies suggest that an increase in GRK2 is detrimental, particularly in cardiovascular disease, but there are some exceptions where it plays a beneficial role in specific tissues or conditions. However, this interplay introduces a novel dimension to understanding cardiac pathologies, as mitochondrial dysfunction is a hallmark of various cardiovascular diseases.

## 6. Conclusions

In summary, GRK2 plays a crucial role in receptor desensitization as well as in cardiovascular health and disease. GRK2 is involved in several physiological processes that preserve cellular homeostasis; notably, GRK2 is a versatile regulator with considerable effects on health and disease. Its canonical function of desensitizing G protein-coupled receptors is extended by its involvement in mitochondrial dynamics and bioenergetics. The crosstalk between GRK2 and mitochondrial dynamics, including oxidative phosphorylation, ROS production, and apoptosis, highlights its potential impact on cardiovascular disease, particularly in heart failure, ischemic damage, and other cardiac dysfunctions. Hence, GRK2 remains a complex protein with a complex interactome. The intricate interplay between GRK2 and mitochondrial function in cardiac tissue reveals an intriguing aspect of cellular regulation. In this context, it is evident that GRK2 holds promise as a potential therapeutic target to attenuate the severity of cardiovascular disease.

Despite the progress made in elucidating the GRK2–mitochondrial axis, it is still unclear what role GRK2 plays in mitochondria, how it acts, and which are the partners inside the mitochondria; therefore, investigating the underlying molecular pathways that regulate GRK2 translocation into mitochondria and its effects on cellular bioenergetics will be crucial for developing targeted therapeutic interventions. The involvement of kinase in mitochondrial dynamics is now evident; thus, it is essential to further investigate the role of GRK2 by trying to answer the question of whether increased kinase levels are always harmful or depend on the type of stimulus (chronic or acute). Furthermore, developing compounds able to move GRK2 within cellular compartments and the identification of protein partners could reveal the role of GRK2 in mitochondria.

Further studies in this area are essential to clarify GRK2’s effects on mitochondrial dynamics and function, paving the way for innovative strategies in cardiovascular medicine.

## Figures and Tables

**Table 1:** The positive and negative effects of GRK2.

GRK2	Research Paper	Key Points
Negative effects	[13,28,34,35,43,69]	- GRK2 increase is associated with cardiac pathologies such as heart failure, ischemia, and hypertension. - Overexpression of GRK2 worsens heart failure and contributes to β-adrenergic receptor desensitization - GRK2 in mitochondria increases oxidative stress, impaired ATP production, and apoptosis. - Inhibition of GRK2 shows therapeutic potential in heart failure by improving mitochondrial function and metabolic processes. - GRK2 phosphorylation at Ser670 following ischemia/reperfusion leads to mitochondrial dysfunction and cell death.
Positive effects	[68,72,75,76]	- Myoblast lines that overexpress GRK2 show increased mitochondrial mass and respiration - In skeletal muscle, GRK2 overexpression promotes mitochondrial respiration, inhibits autophagy, and preserves mitochondrial structure. - In HEK 293, GRK2 interacts with HSP90 to protect against mitochondrial damage during ionizing radiation exposure. - In endothelium, removal of GRK2 increases ROS production and inflammation

## Data Availability

Not applicable.

## Abbreviations

The following abbreviations are used in this manuscript:

CVs	Cardiovascular disease
DRP-1	Dynamin-related protein-1
HF	Heart failure
GPCR	G protein-coupled receptors
GRK	G protein-coupled kinase
OPA I	Optic atrophy 1
MFN-1	Mitofusin 1
MFN-2 PGC-1α ROS	Mitofusin 2 Proliferator-activated receptor gamma coactivator 1-α Reactive oxygen species
